# Meijer and Vloedman’s histochemical demonstration of mitochondrial coupling obeys Lambert–Beer’s law in the myocardium

**DOI:** 10.1007/s00418-018-1716-3

**Published:** 2018-08-29

**Authors:** Eva L. Peters, David Comerford, Frédéric M. Vaz, Willem J. van der Laarse

**Affiliations:** 1 Department of Physiology, Amsterdam Cardiovascular Sciences, Amsterdam UMC, Vrije Universiteit Amsterdam, De Boelelaan 1117, Amsterdam, The Netherlands; 2 Department of Pulmonary Medicine, Amsterdam Cardiovascular Sciences, Amsterdam UMC, Vrije Universiteit Amsterdam, De Boelelaan 1117, Amsterdam, The Netherlands; 3 Department of Clinical Chemistry, Amsterdam Gastroenterology and Metabolism, Laboratory Genetic Metabolic Diseases, Amsterdam UMC, University of Amsterdam, Meibergdreef 9, Amsterdam, The Netherlands

**Keywords:** Pulmonary hypertension, F_1_F_o_ATPase, Oxidative phosphorylation, Histochemistry, Mitochondrial coupling, Heart failure

## Abstract

Uncoupling of mitochondrial proton pumping and adenosine triphosphate (ATP) production lowers mitochondrial efficiency. Current methods to determine mitochondrial efficiency require substantial amounts of tissue and permeabilization or isolation procedures. A simple histochemical method has been described by Meijer and Vloedman (Histochemistry 69:217–232, 1980, 10.1007/BF00489769), but this was not quantitative. We found linear correlations between (1) absorbance and sections thickness and (2) absorbance and incubation time. Because the method obeys Lambert–Beer’s law, we can estimate ATP/O_2_ ratios for healthy and overloaded right-sided rat myocardium. We related mitochondrial efficiency to the ratio between cardiolipin and its precursor phosphatidylglycerol. We found a non-linear relationship between mitochondrial efficiency and this ratio, indicating that lower mitochondrial efficiency as found in experimental pulmonary hypertension may be due to altered composition of the mitochondrial inner membrane. We conclude that the histochemical method of Meijer and Vloedman can be applied to quantify mitochondrial efficiency.

## Introduction

Mitochondrial uncoupling of oxygen consumption and adenosine triphosphate (ATP) resynthesis may play a role in various pathologies. Increased Mg^2+^-stimulated ATPase activity and thus loosely coupled mitochondria, was shown to be a feature of some neuromuscular diseases (Meijer and Vloedman 1980). Also, in cardiomyopathy, mitochondrial dysfunction may play a critical role (Murphy et al. 2016). Decreased right-sided myocardial efficiency of pulmonary hypertensive patients and myocardial preparations of rats (Wong et al. 2010, 2011; Pham et al. 2018) may also be due to uncoupling and a concomitant decrease in the efficiency of mitochondria.

Mitochondrial efficiency measured in intact muscle preparations is 70–80% (corresponding to ATP/O_2_ = 5), indicating that at most 20–30% of the available energy of substrate oxidation is spent on futile ion pumping and heat generation (Lou et al. 2000; Barclay and Widen 2010). The flux of protons crossing the inner membrane without generating ATP by F_1_F_o_ATPase dissipates part of the chemiosmotic proton potential generated by the electron transport chain. Proton permeability of the inner membrane is regulated by uncoupling proteins and hormones and depends on the fatty acid composition of cardiolipin (Hoch 1998). When the futile flux of protons across the mitochondrial inner membrane increases, e.g., due to radical damage to the inner membrane, ATP generation by F_1_F_o_ATPase is disturbed.

Unfortunately, it is often impossible to determine the coupling state of mitochondria in intact preparations because this requires measurements of heat production and/or oxygen consumption and corrections for glycolytic ATP production. Current methods use permeabilized biopsies or isolated mitochondria to determine the coupling state biochemically. The latter methods require relatively large amounts of tissue (50 mg or more). It is also usually unknown how permeabilization or isolation procedures affect the coupling state (Picard et al. 2011). This is especially important because the isolation and permeabilization procedures often need to be optimized differently for test and control tissue.

We studied the possibility to determine the coupling state of mitochondria quantitatively in cryosections of rat myocardial tissue, using the histochemical method as described by Meijer and Vloedman (1980). The method is based on determination of MgATPase activity of F_1_F_o_ATPase operating in the reverse mode, i.e., pumping protons out of the mitochondrial matrix using energy from ATP hydrolysis. We investigated whether this histochemical method obeys Lambert–Beer’s law with respect to the relationship between absorbance and incubation time and the relationship between absorbance and section thickness. Furthermore, we applied the quantification to right ventricular myocardial tissue from control and pulmonary hypertensive rats, and related proton permeability to a marker of cardiolipin metabolism.

## Methods

### Animals

The study was approved by the Animal Experimental Committee of the Vrije Universiteit Amsterdam (Amsterdam, Netherlands). All procedures performed involving animals were in accordance with the guide of the Dutch Research Council for care and use of laboratory animals.

To investigate whether the method obeys Lambert–Beer’s law, four male Wistar rats (body weight ranging from 262 to 341 g at time of experiment, Envigo, The Netherlands) were used. Rats were anesthetized with isoflurane, and hearts were excised and perfused with HEPES-buffered, oxygenated Tyrode [in mM: NaCl 140, KCl 4.7, MgSO_4_·7H_2_O 1.2, NaH_2_PO_4_·H_2_O 2,2,3-butanedione monoxime (BDM) 20, CaCl_2_ 1, HEPES 5, glucose 10, pH = 7.4, 10–15 °C].

To investigate the effect of right ventricular overload on the coupling state of mitochondria in pulmonary hypertensive rats, another four healthy animals were compared with 11 monocrotaline (MCT) treated animals. Tissue was obtained 23 days after subcutaneous MCT injection (60 mg/kg) as described elsewhere (Van Eif et al. 2014). In both cases, the apex and right ventricular free wall were isolated and frozen in liquid nitrogen. F_1_F_o_ATPase was determined within 1 week after collection of the tissue. Tissue was stored at − 80 °C for further analyses.

### Determination of F_1_F_o_ATPase activity

Meijer and Vloedman’s method for complex V activity is based on the determination of F_1_F_o_ATPase activity in a Wachstein–Meisel medium (Meijer and Vloedman 1980). Maximum activity was obtained in the presence of 2,4-dinitrophenol (DNP, Fluka Chemie, Switzerland) which carries protons across the mitochondrial inner membrane. Background activity was measured after inhibition of F_1_F_o_ATPase by oligomycin (Sigma, The Netherlands).

Sections were cut in a Leica CM 1950 cryostat (Nussloch, Germany). For determination of the absorbance as a function of incubation time, sections of 5 µm thickness were incubated for 30 s, 5 min, 10 min or 15 min. To determine the absorbance at different section thicknesses, sections of 2–8 µm thick were cut and incubated for 10 min. The sections were fixed for 2 min on ice in Macrodex (0.9% NaCl, 1% CaCl_2_, 3.6% formalin, 7.7 mM dextran-70) and subsequently washed four times in 0.9% NaCl^+^ 1% CaCl_2_. Sections were then incubated at 37 °C.

The different incubation media were prepared from stock solutions of 0.2 M Tris maleate (pH 7.2), 60 mM Pb(NO_3_)_2_, and 50 mM MgCl_2_. ATP- and oligomycin solutions were freshly made. ATP disodium salt (Sigma, The Netherlands) was dissolved in water and kept on ice until use. Oligomycin was dissolved in ethanol (1 mg/40 µl). Final concentrations were 80 mM Tris maleate, 3.6 mM Pb(NO_3_)_2_, 5 mM MgCl_2_, 1 mM Na_2_ATP, and 25 µM oligomycin and/or 1 mM DNP. The pH was adjusted to 7.2 using 1N NaOH.

To test whether oligomycin inhibition was time-dependent, we pre-incubated sections for 30 min at 37 °C in medium with oligomycin, but without lead nitrate and subsequently incubated in the medium containing both oligomycin and lead nitrate. To determine non-specific binding of lead, sections were incubated without ATP.

After the incubation, sections were washed quickly in four changes of water and developed in 1% Na_2_S, pH 7.5 at room temperature for 1–2 min. Finally, sections were washed again and mounted in glycerin–gelatin.

### Microdensitometry

Images of the sections were captured using NIH image and analyzed using ImageJ (version 1.51u, http://rbs.info.nih.gov) as described previously (Lee-de Groot et al. 1998). None of the images have been manipulated. The absorbance of the final PbS precipitate in the sections was determined at 550 nm in individual myocytes. The absorbances of ten myocytes, cut perpendicularly to the longitudinal axis, were measured in the center of the cell (excluding nuclei). The extinction coefficient of the PbS precipitate at 450 nm is 3788 SD 797 M^−1^·cm^−1^ (Van Noorden and Jonges 1992). At this wavelength, the absorbance is high at 10 min incubation time in 5-µm-thick myocardial sections (> 1 in positive controls), therefore we prefer absorbance measurements at 550 nm. The absorbance of PbS decreases continuously with increasing wavelength > 420 nm (Laborde et al. 1990). The ratio of absorbances at 550 nm over 450 nm measured in myocardial sections was constant up to *A*_450nm_ = 0.6 and equaled 0.54 ± 0.02 [mean ± SD, *n* = 4; confirming results of Laborde et al. (1990)], indicating that the molar extinction coefficient of PbS at 550 nm equals 2046 M^−1^·cm^−1^. We assume that the extinction coefficient at 550 nm with respect to phosphate equals 3069 M^−1^·cm^−1^ since the solubility product of Pb_3_(PO_4_)_2_ is reached before the solubility product of PbHPO_4_ is reached at pH 7.2. A similar value has been reported by Blanco and Sieck (1992).

### Coupling state of mitochondria: quantitative estimate of proton permeability and corresponding ATP/O_2_

It is assumed that F_1_F_o_ATPase activity is negligible in the presence of 25 µM oligomycin (IC_50_ = 0.092 µM, Nesci et al. 2014) and that proton permeability is maximal in the presence of 1 mM 2,4-dinitrophenol (Heytler and Prichard 1962). The estimate of ATP/O_2_ based on energy dissipation due to proton permeability of the inner membrane is calculated assuming the F_1_F_o_ATPase activity is proportional to the proton flux across the inner membrane into the matrix:

$${\text{ATP}}/{{\text{O}}_2}=6.3\;[1 - ({{\text{F}}_1}{{\text{F}}_{\text{o}}}{\text{ATPase}}\;{\text{activity}}/{{\text{F}}_1}{{\text{F}}_{\text{o}}}{\text{ATPase}}\;{\text{activity}}\;{\text{with}}\;{\text{DNP}}),$$

where 6.3 is the theoretical maximum ATP/O_2_ (Kushmerick 1983; Scheffler 2007).

### Cardiolipin and phosphatidylglycerol

Cardiolipin is an essential phospholipid component of the mitochondrial inner membrane. Markers of cardiolipin metabolism were determined in the right-sided myocardium by high-performance liquid chromatography–mass spectrometry (Houtkooper et al. 2009). The ratio of the most abundant phosphatidylglycerol [PG(34:1), a precursor of cardiolipin] and cardiolipin [CL(72.8), containing four linoleic acid fatty acid side-chains] was used as indicator of cardiolipin metabolism. This ratio (PG/CL) is expressed relative to the mean ratio in controls (mean PG/CL is set to 1).

### Statistics

Statistical analyses were performed using GraphPad Prism 7.0 (GraphPad Software, La Jolla, CA, USA). Linear regression lines were fitted through the data and slopes and intercepts were compared using ANOVA. 95% confidence intervals (95% CI) were calculated for the slopes and *y*-intercept. Values are mean ± SD if not indicated otherwise.

## Results and discussion

Pilot experiments indicated that the original fixation time (10 min) was critical in heart muscle. 2-min fixation was sufficient to prevent precipitate covering the section, while maintaining maximal ATPase activity. We did not observe a precipitate covering the section after 2 min of fixation nor the formation of precipitate in the incubation medium, indicating that all phosphate produced in the section precipitated close to the site of formation.

### Absorbance increases linearly with incubation time and section thickness

**Fig. 1:**
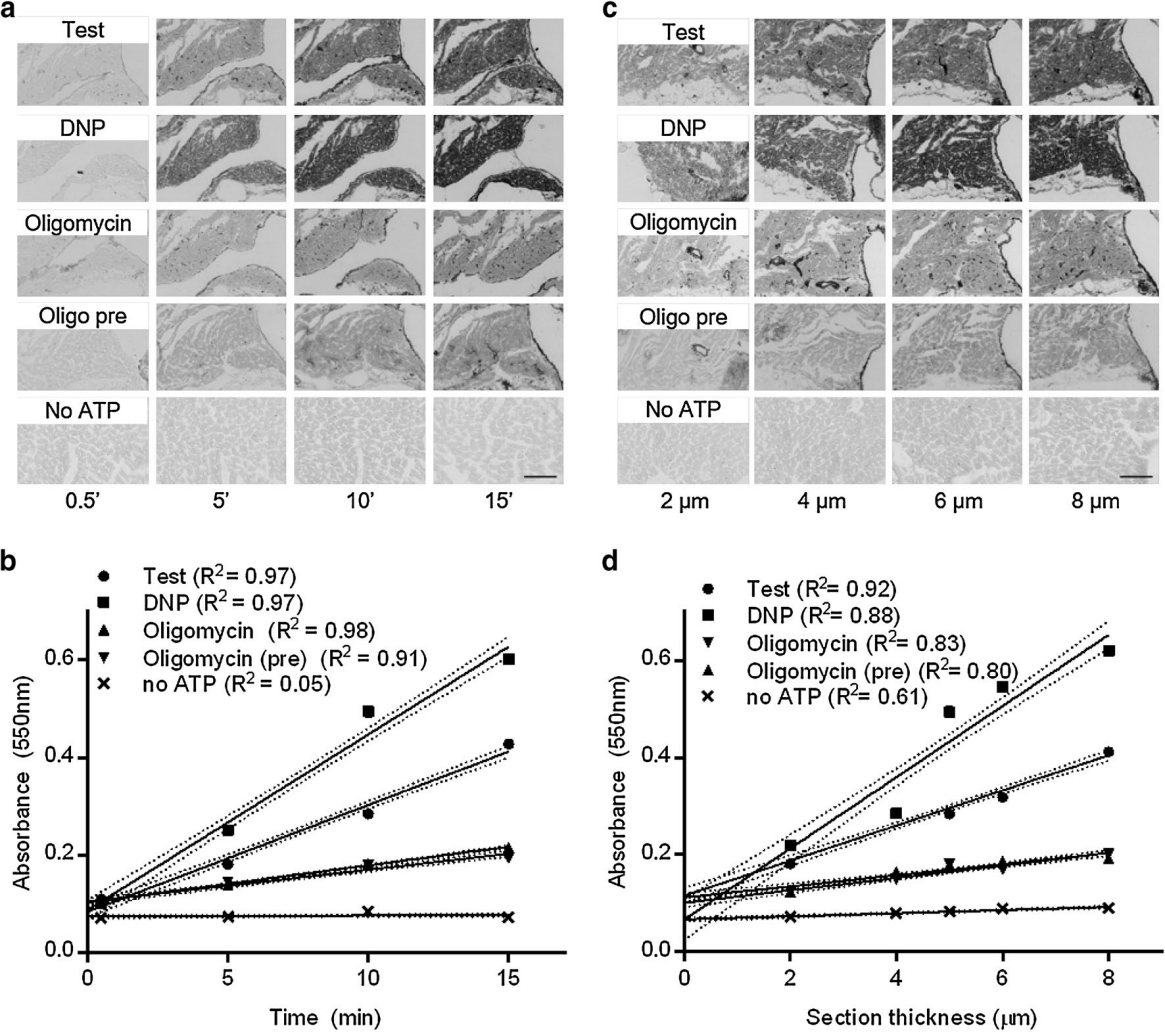
Quantification of time- and sections thickness series. **a** Representative images of serial sections that were incubated for 0.5 min, 5 min, 10 min or 15 min. **b** The absorbance values of the sections shown in **a** were measured and plotted against incubation time. **c** Representative images of serial sections with different thickness, all incubated for 10 min. **d** The absorbance values of the sections shown in **c** were measured and plotted against sections thickness. Data at 5 µm in from **b**, serial sections shown in **a** and **c** are both from the same heart. *R*^2^ values are given in **b** and **d** for the linear regression lines of absorbance with time or sections thickness, respectively. Dotted lines around the regression lines represent 95% confidence intervals. Scale bars represent 100 µm. *DNP* 2,4-dinitrophenol

Figure 1a, b shows the increase in absorbance over time and the images used to measure the absorbance. In all conditions except incubation without ATP, the increase in absorbance was linear with incubation time and significantly different from zero (*p* < 0.0001 for test, DNP, oligomycin and oligomycin + preincubation, *p* = 0.17 for no ATP). However, we observed a *y*-intercept for all conditions (*A*_550_ = 0.094 ± 0.005 on average). The regression lines of the oligomycin incubations with and without preincubation were similar, indicating that oligomycin binding to F_1_F_o_ATPase is fast, and that preincubation is not required. However, part of the ATPase activity is insensitive to oligomycin.

The increase in absorbance with increasing section thickness and the corresponding images are shown in Fig. 1c, d. In all conditions, the increase in absorbance was linear over the measured interval and significantly different from zero (*p* < 0.0001 for all, including no ATP). A *y*-intercept was again present in all conditions (0.099 ± 0.010 on average, 0.066 for no ATP alone). Note that blank absorbance is set just outside the section, indicating that the intercept absorbance in Fig. 1d is due to the interaction of light with the unstained section itself, e.g., due to diffuse light scattering at the cut surfaces of the section. Were it due to PbS absorbance, a proportional relationship was expected in incubations without ATP. The absorbance increase with section thickness of unstained sections is relatively small: 0.0032 absorbance units/μm section thickness. Again, the relationships for oligomycin incubations with and without preincubation are similar, excluding diffusion effects on oligomycin binding.

### Specificity of the reaction

A PbS precipitate is formed even in preparations incubated with oligomycin, indicating that the precipitate is not entirely due to mitochondrial F_1_F_o_ATPase or that oligomycin does not completely inhibit F_1_F_o_ATPase instantaneously. Since oligomycin is present in excess (20 times IC_50_, Nesci et al. 2014) and addition of DNP to oligomycin containing media did not increase the final absorbance after 10 min incubation (result not shown), it is likely that that oligomycin inhibition was complete. Also, both validations (for time and section thickness) showed no differences in the slope or intercept of incubations with oligomycin with or without preincubation. It is thus likely that the time needed for inhibition of F_1_F_o_ATPase is negligible compared to the incubation time. Therefore, we conclude that oligomycin-insensitive ATPases in cardiomyocytes contribute to the absorbance, as discussed by Meijer and Vloedman (1980).

**Fig. 2:**
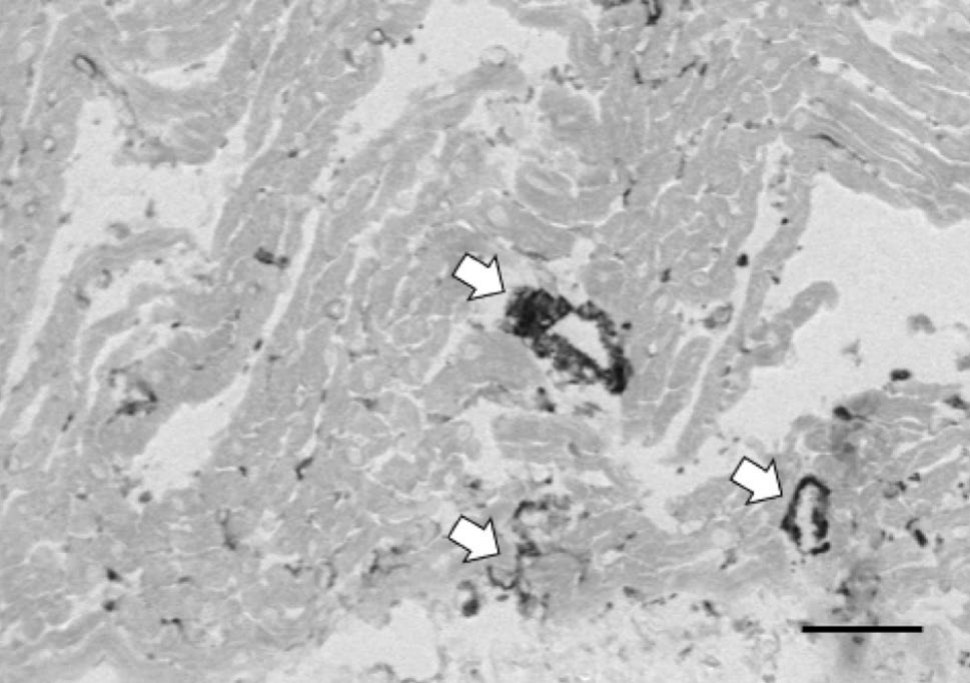
Enlargement of the incubation with oligomycin for 10 min, in a section of 5 µm, as shown in Fig. 1a. White arrows indicate cells with high ATPase activity in vessel walls. Also, larger spots with high ATPase activity are seen, possibly smaller vessels and capillaries. Scale bar represents 50 µm

In addition, these could be myosin ATPase, T-tubular MgATPase (Hidalgo et al. 1983) or ecto-ATPase (Zinchuk et al. 2002). Quantification of these ATPases is beyond the scope of the present study. Ecto-ATPase is mainly located on the outside of the cardiomyocyte sarcolemma and endothelial cells, but has also been detected in T-tubules (Zinchuk et al. 2002). Myosin ATPase does contribute since treatment of sections with 10 μM blebbistatin for 10 min after fixation reduced the absorbance after oligomycin incubation from 0.18 ± 0.02 to 0.14 ± 0.01 (after subtraction of intercept absorbance: from 0.09 ± 0.02 to 0.05 ± 0.01), suggesting that about half of oligomycin-insensitive ATPase activity is myosin ATPase (result not shown). Triton X-100 partly deactivates T-tubular MgATPase (Ebus and Stienen 1996), but cannot be used as inhibitor in the present assay because it solubilizes mitochondrial membranes (Gurtubay et al. 1980). Also, high ATPase activity in vessel walls and endothelial cells is obvious, as shown in Fig. 2. Activity from sarcolemmal ecto-ATPases can easily be excluded from the absorbance measurements, by only measuring the absorbance in the center of the cells.

Thus, accurate determinations of the coupling state using Meijer and Vloedman’s method require a correction for background ATPase activity by subtracting absorbance measured after incubation with oligomycin. Because the intercept is also present in the sections incubated in the presence of oligomycin, subtraction of the absorbance obtained after oligomycin incubation also provides this intercept correction.

Meijer and Vloedman (1980) demonstrated that addition of Pb^2+^ ions to the biochemical assay decreased F_1_F_o_ATPase activity. The inhibition decreased with the amount of tissue in the assay: from 88% inhibition at 2 mg tissue/ml to 23% inhibition at 50 mg tissue/ml homogenization medium, suggesting that inhibition is negligible beyond 200 mg tissue/ml. We conclude that inhibition of the enzyme in the section by Pb^2+^ is negligible in the present study, because tissue density is 1050 mg/ml and because the free Pb^2+^ concentration near the enzyme must be lower than 3.6 mM to maintain the constant flux of lead ions into the section and because Pb^2+^ will at least partly precipitate with phosphate before it reaches the enzyme. It can be calculated using Lambert–Beer’s law that the rate of phosphate precipitation in the section corresponds to 0.34 mM/s in the presence of DNP (Fig. 1b).

### ATP/O_2_ ratios in myocardial control and pulmonary hypertensive rats

**Fig. 3:**
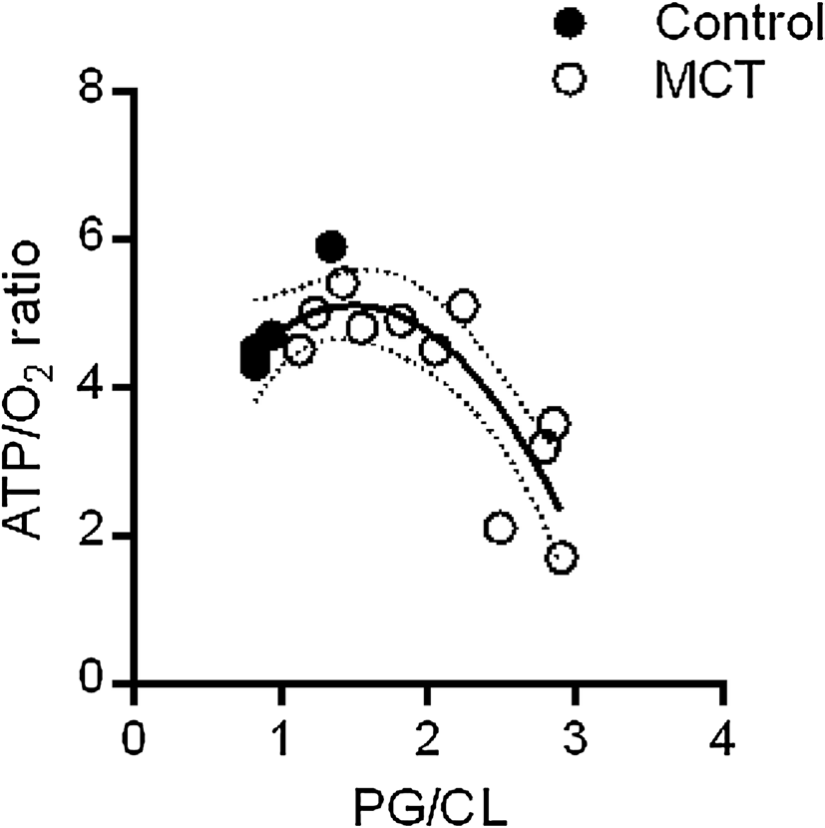
ATP/O_2_ ratios calculated from measured absorbance values, both in control and MCT-induced pulmonary hypertensive rats. A second-order polynomial was fitted through the data (best fit curve ATP/O_2_ = − 1.4 (PG/CL)^2^ + 4.18 (PG/CL) + 1.99, *R*^2^ = 0.69). *PG* phosphatidylglycerol, *CL* cardiolipin

After correction, the absorbance data in test and positive controls obey Lambert–Beer’s law and thus, it is possible to estimate the effect of proton permeability on ATP/O_2_. The result is shown in Fig. 3 where the relationship between the ATP/O_2_ ratio calculated from proton permeability is plotted against a marker of cardiolipin metabolism, the PG/CL ratio. Paired determinations in the right ventricular free walls of healthy and pulmonary hypertensive rats show a normal ATP/O_2_ = 5 in healthy hearts but a non-linearly decreasing coupling ratio with increasing PG/CL ratio. Low ATP/O_2_ ratios of around two have severe energetic consequences. These results suggest that mitochondrial proton leak could be an important contributor to reduced myocardial efficiency in chronic heart failure. The mechanism behind the relationship in Fig. 3 requires further study. The present histochemical method can be applied to myocardial samples smaller than a milligram, allowing for diagnostic tests of mitochondrial function in myocardial biopsies.
